# Foreign Bodies on the Cornea

**Published:** 1861-03

**Authors:** E. L. Holmes

**Affiliations:** Chicago


### FOREIGN BODIES ON THE CORNEA.

BY E. L. HOLMES, M. D., of Chicago.

This subject has been considered of so much importance, that a chapter of greater or less length is devoted to it in almost every systematic work upon diseases of the eye. Although the works of eminent oculists are open to the profession, and an article upon the subject may perhaps seem to the readers of the *Journal* uncalled for, there are several reasons, why we wish to offer a few remarks for the consideration of those who attempt to remove foreign substances from the cornea.

We have met with several cases in which physicians had failed to detect the presence of minute particles embedded in the cornea, and had treated the eye with irritating collyria as in catarrhal ophthalmia. We have also met with three cases, in which an eye had been ruined by awkward attempts to remove particles of steel from the cornea. Nearly all our standard works on ophthalmology allude to cases of this kind. In every manufacturing community the number of patients who suffer from this little accident, and who apply to physicians for aid, must be quite large. We have reason to believe that the aggregate number of those who have lost an eye from the carelessness or inefficiency of physicians in treating these cases, is larger than many might at first suppose. Moreover there is a common idea among patients and even among physicians, that the surgical treatment of such injuries is too simple to require particular practice, or even a passing notice. While we are far from wishing to claim any undue importance for this subject, we deem the reasons above given sufficient grounds for introducing the subject at the present time.

Particles of almost every variety of hard substance have been found embedded more or less deeply in the cornea. There is probably no more frequent cause of this accident, however, than the forcible abrasion of steel in cutting stone or metals. Mechanics, stone cutters and masons are especially liable to this injury. Farmers, also, during harvest, suffer occasionally from the pressure of the minute barbs from grain becoming fixed upon the cornea. In fact every one is liable, in an infinite variety of ways, to this accident.

Whenever a foreign substance, however minute, is lodged upon the cornea, the patient usually experiences more or less pain, especially in the act of winking; the conjunctiva becomes congested, its enlarged vessels encroaching upon the cornea, the eye becomes exceedingly sensitive to light; ulceration commences around the foreign body, which is sometimes thus cast off. The pain and congestion are variable in degree in different cases. Difference of symtoms in cases apparently similar is often unaccountable. From the first our patient may suffer from violent pain and inflammation, which, unless the object is removed, may run its course with great rapidity, totally disorganizing one tissue after another; and yet the primary injury would seem less severe than in another case, where a larger particle, more deeply embedded in the cornea, would produce comparatively little irritation, after the lapse of several days, or even weeks.

A careful examination is sometimes necessary to detect particles upon the cornea, especially when they are concealed from view by peculiarity of color or pus and mucous collected around them. They are frequently so minute that practitioners with ordinary powers of vision are unable to discover them. Larger objects even sometimes escape notice. We have seen a patient with quite a large fragment of a common "bird seed" on the cornea, examined by several physicians, who failed to make a correct diagnosis. The concave surface of the fragment had become firmly attached to the tissues at the union of the cornea and sclerotic. It was almost wholly covered with pus and mucous and was surrounded with a circle of enlarged vessels. The eye certainly presented in almost every respect the appearance of phlyctenular ophthalmia and was so diagnosticated by the physicians present. They were much astonished, however, when the oculist in charge called for an instrument and with it quietly removed the offending body.

A satisfactory examination can usually be best made when the patient sits facing a window—the surgeon being between the patient and light. The light, however, should not be too brilliant. By directing the patient to rotate the eye in different directions the light will finally fall upon the cornea in such a way that a clear view of the object may be obtained. The same care is often requisite in examining certain minute and transparent ulcerations of the cornea. We call particular attention to this point, because physicians are often unable to detect either minute particles on the cornea or ulcerations, simply from failure in placing the patient in a suitable light.

The little operation necessary in these cases, trifling as it may appear, is one which in many instances should not be undertaken by any physician, unless he possesses keen vision and a steady hand. The patient should be placed in a good light, the head resting against a firm support. We often prefer to stand behind the patient seated in a chair facing a window. In this way the head can be securely supported, while the light will fall upon the cornea most favorably for the operation. If the accident is recent and the foreign substance is not too deeply embedded in the cornea, it can be readily removed with the assistance of a bit of stiff paper or cloth folded in a convenient form. We often remove such substances with a slender piece of dry pine, sharply pointed at one end. A cataract needle can be used advantageously for the same purpose. A good magnet will sometimes remove particles of iron and steel.

But very frequently the removal of these particles will be found by no means so easy. They are often held with surprising tenacity deep in the cornea. A sudden "dive" at them with any sharp instrument will not be sufficient. The lids must be kept open and the globe held with the fingers of one hand, while with the other a cataract needle or some similar instrument is employed to remove the foreign body. It must be deliberately cut from its position. "It is sometimes necessary to pass the point of the instrument under the particle of iron or stone so as to lift it out of the cornea: and so firmly is the foreign body grasped in many instances that even this plan will not succeed, unless the portion of cornea external to the foreign particle is first divided and then pressure applied in the way described." It should be remembered that in some cases it is perhaps impossible to remove particles deeply seated in the cornea without perforating this membrane. We have seen so skilful and delicate an operator on the eye as M. Desmarres, of Paris, perforate the cornea after making several attempts to remove a particle of steel, although the patient sat with wonderful fortitude through the painful operation. Fortunately, the wound healed without injury to the eye.

In all cases where the simple means first described are insufficient to detach foreign bodies from the cornea, we deem it safer to perform the operation while the patient is under the influence of some anæsthetic, since few patients have perfect control over the eye when the cornea is touched, however slightly, with any cutting instrument. Every one must appreciate this difficulty on the part of patients, who has made many attempts to *enucleate*, as it were, foreign bodies from the cornea.

Occasionally fine fragments of metal will be so deeply implanted in the cornea, that great dexterity and care are required to avoid pushing them through into the anterior chamber. In such extreme cases, M. Desmarres recommends the use of a very small lance-shaped knife, the point of which is to be passed obliquely through the cornea and held in such a manner that the flat surface in the anterior chamber shall rest against the concave surface of the cornea behind the object. With this precaution a cataract needle can be used with no fear of the accident just mentioned.

It should be remembered that after the removal of certain metalic particles, an oxide sometimes remains incorporated in the cicatrix. This oxide should always, if possible, be cleared from the wound with the suitable instrument, since otherwise an opacity may render vision more or less obscure.

The subsequent treatment of these cases is almost always quite simple. Usually nothing is required, when there is merely a minute abrasion of the epithelium. In severer cases it is well to keep the eye closed for a short period, and to apply cooling lotions externally. But it should be borne in mind that, even with patients apparently in perfect health, the most minute and superficial abrasions of the cornea will some. times not heal for weeks or even months. We have seen patients who were obliged to shade an eye from ordinary light for several weeks and yet there was no congestion of the tissues, and few physicians would be able to detect the very minute ulceration produced by the foreign body. We have always found mild, stimulating collyria and rest with closure of the eye to succeed finally in accomplishing a cure. Wherever the injury of the cornea is very deep it may become necessary to treat the case with more care, as in cases of serious ulceration of the same part.

